# Comparison of Brachial Compression Versus Ulnar Compression on Radial Artery Diameter: A Randomized Controlled Trial

**DOI:** 10.1155/2024/9965794

**Published:** 2024-11-14

**Authors:** Fatemeh Bahrami, Shayan Mirshafiee, Pejman Mansouri, Mohammadreza Eftekhari, Mohammad Vahidi, Fateme Baharvand, Ehsan Moradi Farsani, Hamed Vahidi

**Affiliations:** ^1^Department of Cardiology, School of Medicine, Tehran University of Medical Sciences, Tehran, Iran; ^2^Tehran Heart Center, Tehran University of Medical Sciences, Tehran, Iran; ^3^Department of Radiology, Shahid Beheshti University of Medical Sciences, Tehran, Iran; ^4^Cardiovascular Diseases Research Center, Department of Cardiology, Heshmat Hospital, School of Medicine, Guilan University of Medical Sciences, Rasht, Iran

**Keywords:** brachial compression, flow-mediated dilation, transradial coronary angiography, ulnar compression

## Abstract

**Objectives:** This study is aimed at comparing the effectiveness of ulnar compression and brachial compression in inducing radial artery dilatation.

**Methods:** This randomized crossover study included 30 patients undergoing elective diagnostic transradial coronary angiography. Ulnar compression and brachial compression maneuvers were performed in two groups. Radial artery diameter and cross-sectional area were measured at baseline and remeasured every 30 s (up to 2 min) after the interventions.

**Results:** Both ulnar compression and brachial compression maneuvers successfully increased radial artery diameter for up to 60 s following the interventions. There were no statistically significant differences between the two groups after adjusting for baseline measurements. However, each treatment group showed a significant increase in indicators up to 60 s, followed by a subsequent decrease. The maximum radial artery diameter occurred at 60 s after the removal of compression in both groups.

**Conclusion:** Ulnar compression and brachial compression maneuvers demonstrated effectiveness in inducing radial artery dilation for a limited duration. These maneuvers may reduce the occurrence of access failure during radial artery cannulation. No significant differences were observed between the two maneuvers, indicating that they can be used interchangeably based on clinician preference. So, because the ulnar compression is simpler and more feasible for the patients, it can be considered instead of brachial compression.

**Trial Registration:** IRCT20230209057372N1.

## 1. Introduction

Cardiovascular diseases (CVDs) continue to be the leading cause of illness and death worldwide. As a result, there is a growing global trend towards increased coronary evaluation, highlighting the importance of adopting more patient-friendly diagnostic approaches for individuals undergoing coronary angiography (CAG). [1–3] The transradial approach (TRA) is the preferred access for cardiac catheterization either in elective or urgent procedures because of lower complications and hospital stay [4]. The most frequent limitation of this approach is radial artery (RA) spasm, which reduces the procedure's success rate and causes intense pain in the forearm [5, 6]. More puncture attempts and pain during RA cannulation increase the probability of spasms during the procedure [7]. Several factors have been identified as predictors of RA access failure, including a narrow radial artery diameter (RAD), challenging access, operators lacking experience, and the presence of peripheral arterial disease [8].

Routinely, antispasmolytic cocktails including vasodilator agents like 5 mg of verapamil and or 200 *μ*g of nitroglycerine are administered through the arterial sheath which can effectively reduce spasm rate from 12% to 4% compared to placebo, but they have unwanted side effects like bradycardia and hypotension [5, 9, 10].

One of the main reasons for access site crossover is RA spasm, which can be prevented effectively by drugs or maneuvers causing RA [11, 12] Measuring RA diameter and cross-sectional area by ultrasound technique following the induction of hyperemia has been studied. Ipsilateral ulnar artery compression (UAC) for a minute can increase RA diameter significantly which decreases after a minute from stopping compression [13]. This inexpensive technique can also reduce the rate of RA occlusion after catheterizations [14]. Another way to induce RA dilation is wrapping the sphygmomanometer cuff around the arm for 5 min, inflating it rapidly about 50 mmHg above systolic blood pressure (SBP), and then deflating it quickly. This can also fasten spasm recovery which happens during procedures [15].

We decided to compare the effect of these two methods, ulnar compression versus brachial compression, on the dilation of the RA.

## 2. Material and Methods

This cross-over randomized, controlled, interventional, study was conducted in the Imam Khomeini Cardiovascular Medical and Research Center, a tertiary referral center affiliated to Tehran University of Medical Sciences. The Ethics Committee approved the study protocol based on code number “IR.TUMS.IKHC.REC.1401.310.” Furthermore, it has been registered in the Iranian Registry of Clinical Trials. Before enrolling in the study, the researchers informed the patients and their families about the study protocol and assured them that their personal information would be kept confidential. The patients and their families then provided written consent.

Participants were included if they were aged over 18 and candidates for elective diagnostic transradial CAG. Our exclusion criteria were acute coronary syndrome or need for primary percutaneous coronary intervention (PCI), history of transbrachial or trans-RA catheterization, use of vasoactive drugs, chronic kidney disease (CKD), other rhythm than in sinus rhythm, hemorrhagic disorders or coagulopathies, and severe systematic disorders (e.g., sepsis), hemodynamically unstable patients, and negative Allen's test. Allen's test was done for all of the patients before the procedure.

The study used a randomized crossover design to assign eligible subjects to two groups. Random Allocation Software by which each patient was provided with a particular number allocated him/her to one of the groups. The first group received ulnar compression maneuver (by inflation of TR Band arterial compression device around the patient's wrist via inflator syringe with 15 mL of air and confirmation of ulnar artery flow interruption by Doppler's ultrasound) at 8:00–10:00 AM and brachial compression maneuver (by inflation of a sphygmomanometer cuff that wrapped around the arm, 30 mmHg above SBP for 5 min and then deflated) at 4:00–6:00 PM. The second group received the opposite schedule.

An expert interventional cardiologist (V.H.) performed all the ultrasound measurements in semidarkened room using a VIVID 7 ultrasound system (multi-frequency linear transducer, GE Healthcare, Madison, WI, United States) at 2 cm above the styloid process. RA diameter and flow were acquired at baseline and remeasured every 30 s (up to 2 min) after the end of the mentioned maneuvers (ulnar compression/upper arm compression). The primary outcome was investigating the difference in the effect of these two maneuvers on the RA diameter.

Data were statistically analyzed using SPSS 25 for Windows (SPSS Inc., Chicago, IL, United States). Results are shown as mean ± standard deviation (SD) and as numbers and percentages for categorical data. Categorical variables were analyzed using the *χ*^2^ test, and continuous variables were analyzed using the Student's *t*-test. A generalized linear mixed effects model was used to analyze changes between treatment groups. The difference between the start of treatment and carry-over effect was treated by adjusting baseline measurement as covariance. Repeated measurement ANOVA and Bonferroni's test were used to check the changes in each group. *p* value less than 0.05 was considered statistically significant.

## 3. Results

**Figure 1:**
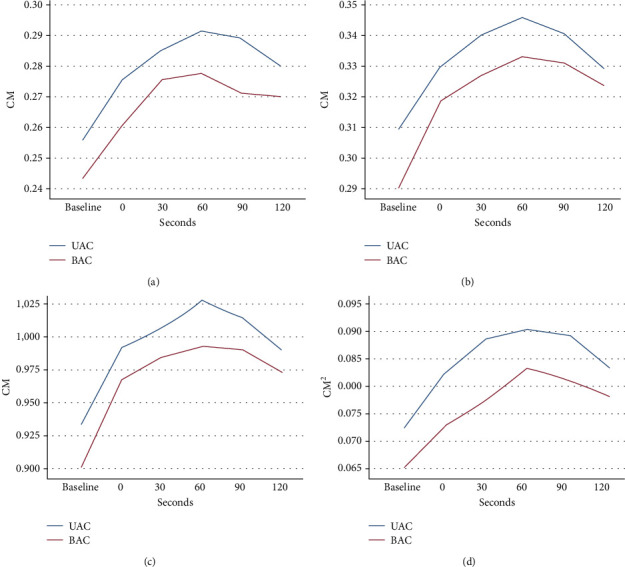
The average of four measured radial artery diameters before each intervention and after the end of compression in both groups: (a) D1: longitudinal diameter, (b) transverse diameter, (c) C: circumstance, and (d) area. UAC, ulnar artery compression; BAC, brachial artery compression.

From July 2023 to August 2023, a total of 30 patients were randomly allocated to two groups, with 11 patients (36.7%) being women. The average age of the participants was 51.3 years, with a standard deviation of 11.5 years. The baseline characteristics of the patients are presented in Table 1.  Table 2 and Figure 1 show the average of four measured RA diameters before each intervention and after the end of compression in both groups. There was no relationship between baseline measurements and time of doing maneuvers. Statistical analysis revealed that after adjusting for baseline measurements, there was no statistically significant difference between the two groups (*p* value = 1.000). However, each treatment group was able to increase the indicators up to 60 s, with statistically significant differences (*p* value < 0.01), before experiencing a subsequent decrease, although it did not reach the initial level until the 120th second. Our results demonstrate that the maximum of the radial diameter happened at 60 s after the removal of the compression in both groups.

## 4. Discussion

The TRA is widely used for cardiac catheterization due to its favorable outcomes and reduced complications compared to the transfemoral approach. However, RA spasm remains a common limitation of TRA, leading to decreased procedural success rates and patient discomfort. Antispasmolytic cocktails are routinely administered to mitigate RA spasms, but they often come with unwanted side effects. Therefore, alternative methods for preventing RA spasms, by means of reduction in access site failure rate such as drug administration or mechanical maneuvers, have been explored.

In this study, we compared two different methods of RA dilation, ulnar compression maneuver, and brachial compression maneuver to evaluate their possible effectiveness in RA cannulation and therefore preventing RA spasm during transradial CAG. Our results demonstrated that both maneuvers were successful in increasing diameter and area for up to 60 s following the interventions.

The difficulty of achieving successful punctures is heightened by hypotension and the presence of small-sized, deeply located radial arteries (RAs), particularly for operators with less experience. Additional challenges arise from preprocedural anxiety and pain during local anesthetic injection or puncture attempts, which can induce vasospasm. Administration of sedatives to alleviate anxiety may further complicate puncture by causing hypotension and the loss of RA pulse. With each unsuccessful attempt, the RA becomes increasingly susceptible to spasm. The primary factor influencing successful access is the size of the RA, with similar findings reported in previous studies [16]. The efficacy of brachial artery compression on RA dilation and treating puncture–induced RA spasm is well known and has been frequently used for many years, but UAC has not been investigated as much [17].

In a recent study, the effectiveness of transient ulnar compression was evaluated in patients undergoing transradial CAG in comparison to a standard protocol group without compression. The ulnar compression group demonstrated a significant reduction in the number of attempts for radial access, accompanied by a substantial increase in the rate of first-pass success. Additionally, the access time was significantly shorter in the ulnar compression group. Furthermore, the ulnar compression group exhibited a lower proportion of difficult procedures compared to the standard group. However, there were no significant differences observed in the failure rates of sheath insertion and puncture between the two groups. These findings highlight the potential benefits of employing ulnar compression as an adjunctive technique to improve the success and efficiency of transradial CAG procedures [18].

In a separate research endeavor delving into the consequences of UAC on RAD, the study methodically assessed RAD at different intervals without utilizing a control group. The ulnar artery underwent compression for a precise duration of 1 min. The analysis revealed a substantial difference in RAD between diabetic and nondiabetic patients, with diabetic individuals presenting a significantly smaller RAD. Additionally, women demonstrated a smaller RAD in comparison to men. Subsequent to the UAC intervention, there was a marked increase in RAD compared to the baseline measurement (2.45 ± 0.41 mm to 2.62 ± 0.41 mm, *p* < 0.001. Nevertheless, following the discontinuation of UAC, there was a decrease in RAD, with a significant reduction observed at 1 min post-UAC (2.62 ± 0.41 mm vs. 2.55 ± 0.40 mm, *p* < 0.001), although it remained significantly larger than the baseline (*p* < 0.001). These results underscore the dynamic nature of RA diameter changes following UAC, indicating potential implications for optimizing transradial procedures in specific patient subsets [13].

As the RA has *α*-1 adrenoceptor dominancy rather than *β*-adrenoceptors, circulating catecholamines, which are released by pain, will mostly activate *α*-1 adrenoceptors and therefore cause spasm [19]. So, it seemed that UAC which is more painless may be more successful in RA dilation, but we realized that this factor does not have clinical significance, and both maneuvers increase the diameter by the same proportion and interestingly at the same time.

## 5. Limitations

This study exclusively evaluated changes in diameter, indicating that future research is required to ascertain whether these changes genuinely enhance clinical puncture success rates.

## 6. Conclusion

In conclusion, both ulnar compression maneuver and upper arm compression maneuver significantly increased flow-mediated dilation of RA. No significant differences were observed between the two maneuvers, indicating that they can be used interchangeably based on clinician preference. So, because the ulnar compression is simpler and less painful for the patients, it can performed instead of brachial compression. Further research is needed to validate these findings and investigate their long-term implications and their possible effect on the reduction of RA spasm and access failure.

## 7. Transparency Statement

The lead author affirms that this manuscript is an honest, accurate, and transparent account of the study being.

## Figures and Tables

**Table 1:** Baseline characteristics of patients.

**Variable**	
Female N (%)	11(36.7%)
Age (mean ± SD)	51.3 ± 11.5
Diabetes mellitus	6(20%)
Hypertension (%)	15 (50%)
Ischemic heart disease (%)	7 (23.3%)
Dyslipidemia (%)	4 (13.3%)
Smoker (%)	14 (46.7%)
Opium consumption (%)	4 (13.3%)
Systolic blood pressure (mean ± SD)	127.7 ± 15.5
Diastolic blood pressure (mean ± SD)	81.6 ± 9.7

Abbreviation: SD, standard deviation.

**Table 2:** Measurements of the radial artery at baseline and after the compression (0, 30, 60, 90, and 120 s).

**Treatment**	**Variable**	**Time**						**p** ** value**
		**Baseline**	**0 s**	**30 s**	**60 s**	**90 s**	**120 s**	
Ulnar compression	D1 (centimeters)	0.25 ± 0.04	0.27 ± 0.05	0.28 ± 0.05	0.29 ± 0.05	0.29 ± 0.05	0.28 ± 0.05	0.009
	D2 (centimeters)	0.30 ± 0.06	0.32 ± 0.05	0.34 ± 0.05	0.34 ± 0.06	0.34 ± 0.06	0.33 ± 0.05	0.008
	C (centimeterss	0.93 ± 0.16	0.99 ± 0.14	1.01 ± 0.14	1.03 ± 0.16	1.01 ± 0.16	0.99 ± 0.15	≤ 0.001
	Area (square centimeters)	0.07 ± 0.02	0.08 ± 0.02	0.09 ± 0.02	0.09 ± 0.03	0.09 ± 0.02	0.08 ± 0.02	≤0.001

Upper arm compression	D1 (centimeters)	0.24 ± 0.03	0.26 ± 0.04	0.27 ± 0.05	0.27 ± 0.05	0.27 ± 0.05	0.27 ± 0.05	≤ 0.001
	D2 (centimeters)	0.29 ± 0.05	0.31 ± 0.04	0.32 ± 0.06	0.33 ± 0.05	0.33 ± 0.05	0.32 ± 0.05	≤ 0.001
	C (centimeters)	0.90 ± 0.13	0.97 ± 0.11	0.98 ± 0.14	0.99 ± 0.15	0.99 ± 0.14	0.97 ± 0.14	≤ 0.001
	Area (square centimeters)	0.06 ± 0.01	0.07 ± 0.01	0.07 ± 0.02	0.08 ± 0.02	0.08 ± 0.02	0.07 ± 0.02	≤ 0.001

*Note:* D1: longitudinal diameter; D2: transverse diameter.

Abbreviation: C, circumference.

## Data Availability

The data supporting the findings of this study can be requested from the corresponding author.
